# Psychometric properties of the Norwegian version of the Evidence-Based Practice Attitude Scale (EBPAS): to measure implementation readiness

**DOI:** 10.1186/s12961-016-0114-3

**Published:** 2016-06-17

**Authors:** Karina M. Egeland, Torleif Ruud, Terje Ogden, Jonas Christoffer Lindstrøm, Kristin Sverdvik Heiervang

**Affiliations:** Akershus University Hospital, Division of Mental Health Services, Sykehusveien 25, 1478 Lørenskog, Norway; Institute of Clinical Medicine, University of Oslo, Oslo, Norway; Norwegian Center for Child Behavioral Development, Essendropsgate 3, 0368 Oslo, Norway; Institute of Psychology, University of Oslo, Essendropsgate 3, 0368 Oslo, Norway; Helse Sør-Øst Health Services Research Centre, Akershus University Hospital, Sykehusveien 25, 1478 Lørenskog, Norway

**Keywords:** Implementation, Evidence-based practice, Readiness, Practitioner selection, Mental health services

## Abstract

**Background:**

Attitudes can be a precursor to the decision of whether or not to try a new practice. In order to tailor the implementation of evidence-based practices (EBPs) in mental health settings, we must first consider practitioner attitudes towards EBP adoption. To assess these attitudes, the Evidence-Based Practice Attitude Scale (EBPAS) was developed. The purpose of this study was to investigate the psychometric properties of the Norwegian version of the EBPAS, and to examine differences in attitudes towards implementing EBPs among mental health practitioners.

**Methods:**

The EBPAS was translated into Norwegian and administered to 294 practitioners from seven primary and 22 specialized mental care units within a defined geographical area of Norway.

**Results:**

The EBPAS showed good psychometric properties. The less clinical experience the practitioner had, the more positive their attitude toward EBPs. Primary care practitioners reported more positive attitudes towards implementing EBPs that were required of them than specialized care practitioners.

**Conclusions:**

The Norwegian version of the EBPAS is a promising tool for measuring implementation readiness in mental health services, and can be used in clinical practice to tailor implementation efforts.

**Trial registration:**

The study was approved by the regional committees for medical and health research ethics [REK 2013/2035] on 25^th^ of May, 2014.

## Background

Despite the increased focus on implementing evidence-based practices (EBPs) in mental health, it is still considered a struggle to bring such practices to the service users who would benefit from them [1]. EBP is the integration of the best available research with clinical expertise in the context of client characteristics, culture and preferences [2]. Multiple factors influence the implementation of EBP in real-world settings, including practitioners’ attitudes. Some studies have shown that practitioners’ general attitudes toward EBP can hinder or facilitate their decision of whether or not to try a new intervention [3, 4]. This is in accordance with Ajzen’s [5] theory of planned behaviour, which indicates that attitudes (a person’s positive or negative evaluation of performing the behaviour) along with subjective norms (a person’s perception of the social pressures put on them to perform or not perform the behaviour) and perceived behavioural control (a person’s confidence in their ability to perform a behaviour, often referred to as self-efficacy) influence one another, and together shape a person’s intentions and behaviour. Ajzen proposed that, as a general rule, the more favourable the attitudes, subjective norms, and perceived behavioural control, the stronger an individual’s intention to engage in the behaviour in question. However, this will be expressed only if the person decides at will to perform it or not. Even then, it may not be performed because of non-motivational factors such as the perceived availability of requisite opportunities and resources [6].

Assessing attitudes toward EBPs prior to implementation may give useful information about practitioners’ readiness to adopt a new intervention. Aarons [7] developed the Evidence-Based Practice Attitude Scale (EBPAS) to assess mental health practitioners’ attitudes towards adopting EBP in mental health settings. The EBPAS assesses four dimensions: the intuitive appeal of EBPs, the likelihood of adopting an EBP given the requirements to do so, openness to new practices, and perceived divergence between research-based interventions and current practice [8]. The higher the score, the more positive the attitude toward EBPs, except for the divergence scale, which is scored in reverse. Previous studies have suggested adequate internal consistency for the EBPAS total score (α = 0.79–0.77) and good internal consistency for the subscale scores (α = 0.93–0.74), except for the divergence scale ranging somewhat lower across studies (α = 0.66–0.56) [7, 9–12]. The studies also supported or partly supported the construct validity by finding acceptable model–data fit for confirmatory factor analysis (CFA) models, including acceptable fit indices for both a first-order structure and a higher order global structure. The content validity of the scale has been supported [12]. The criterion validity has shown mixed findings; some have found it to predict adoption or use of EBP [13–16], and others have not found a relation between EBPAS scores and EBP behaviours [17, 18]. Studies that have examined sensitivity to change have reported little variation of EBPAS scores over time [19, 20].

In search of better strategies to implement EBP in health services, researchers have used the EBPAS to examine factors that appear to affect practitioners’ attitudes towards EBPs. In terms of professional experience, practitioners with longer experience have scored lower on appeal, openness, requirements and the total EBPAS [10, 12, 21]. Mental health practitioners’ experiences were associated with higher scores on divergence in one study [22], and lower in another [12]. Practitioners with higher education scored lower on requirements, and higher on appeal and the EBPAS total [12, 22–24]. Results concerning differences by sex and age have been inconsistent; some studies found no differences by sex [7, 10], but others found women to score higher on appeal, requirements and the EBPAS total [12, 23]. Concerning age, some studies found younger practitioners to score higher on the EBPAS total [10, 17]. Others have found older practitioners to score higher on requirements and openness scores [12, 25], but that they also scored higher on divergence [25]. Attitudes have also been associated with organizational characteristics. For example, practitioners in less bureaucratic programs [7], more proficient organizational cultures and less stressful climates [21] have been shown to favour EBPs.

Except for one study among 966 Norwegian physicians that showed limited knowledge and experience of evidence-based medicine, but a positive attitude toward the concept [26], little is known about health practitioners’ attitudes in Norway. The Norwegian mental healthcare system is semi-decentralized [27]. Primary care is organized by the municipalities. Their political mandate is to deliver mental health services to users with all types of mental illnesses and close to where they live. Practitioners have a primary background in a mental health discipline or primary training in nursing, social education or social work, for example, with supplemental training in a mental health discipline. They offer individual outpatient psychotherapy, psychotropic medication management, and home visits. In most municipalities, there are few or no psychologists or psychiatrists. Because of large differences in size and organization among the municipalities, primary care practitioners are, to a large extent, at liberty to decide the content and organization of their services [27], which makes them more flexible and subject to fewer governmental restrictions than specialized care practitioners.

The government is responsible for specialized care, which is administered by four regional health authorities, each responsible for several health authorities that include mental health divisions consisting of both inpatient and outpatient services. To a great extent, specialized care consists of specialists in psychiatric nursing, psychiatry and psychology. Their main task is to diagnose and give specialized psychotherapeutic and medical treatment to service users with severe mental illnesses. Specialized care is subject to more formalized guidelines that are developed by the Norwegian Directorate of Health [28, 29].

To our knowledge, no other study to date has examined Norwegian mental health practitioners’ attitudes using the EBPAS. The purpose of this study was to investigate the psychometric properties of the Norwegian version of the EBPAS, and to examine differences in attitudes towards EBP among mental health practitioners. Understanding practitioners’ attitudes can give insights about their readiness to implement EBPs in their services, and contribute to the tailoring of better implementation strategies that strengthen EBP uptake. Specifically, we investigated the following research questions:

1. Does the Norwegian version of the EBPAS show satisfactory reliability and supported factor structure compared to the original version?
2. Does the Norwegian sample differ from a nationwide sample in the United States on attitudes among mental health service practitioners?
3. What role does practitioners’ experience play as a predictor for attitudes towards EBP in the Norwegian sample?
4. Are there differences in attitudes between practitioners in primary and specialized mental healthcare in the Norwegian sample?

## Methods

### Participants and procedure

The present cross-sectional study took place in the southern part of Norway, which represents the largest catchment area of a health authority. The regional committees for medical and health research ethics approved the study [REK 2013/2035]. Data were collected from October 2013 to May 2014. Seven primary care units in one geographical area were asked to participate, and they all accepted. Additionally, 30 of 36 eligible specialized care units were asked to participate in the survey. When 22 of the units had responded positively, it was considered a sufficient sample because these units represented all types of clinics in all departments in adult psychiatric care within the hospital trust. In 20 of the units, the first author made an appointment with the whole team of practitioners. The pen and paper survey was distributed and collected at these meetings (87.5% response rate). In nine of the units, the pen and paper survey was distributed to an employee who administered the survey to the other practitioners in the unit. The employee collected the survey in an envelope and sent it to the first author (78.4% response rate). Of 375 employees asked to participate, 315 (84%) accepted the invitation. Because of missing data on the EBPAS total scale, 21 surveys were excluded, leaving 294 respondents.

Of the participants (N = 294), 71.4% (n *=* 210) were from specialized inpatient and outpatient care units, and the remaining 28.6% (n = 84) were from primary care units. As shown in Table 1, practitioners in primary and specialized care differed significantly in level of education (χ^2^ test, *P* < 0.001) and discipline (χ^2^ test, *P* < 0.001). This makes sense, as primary care participants mostly held bachelor’s degrees in nursing/social education or social work, whereas specialized care participants mostly held master’s degrees in psychology, nursing/social education, or medicine.

**Table 1:** Participant demographic information

	Primary care (n = 84)		Specialized care (n = 210)		Total (N = 294)	
Characteristic	M	(SD)	M	(SD)	M	(SD)
Age	46.6	(10.1)	44.3	(11.1)	44.9	(10.8)
Years of experience	13.9	(8.7)	13.9	(10.3)	13.9	(9.9)
	N	(%)	N	(%)	N	(%)
Female	59	(70.2)	152	(72.4)	211	(71.8)
Education level^a^						
Lower	18	(21.4)	1	(0.5)	19	(6.5)
Bachelor	58	(69.0)	58	(27.6)	116	(39.5)
Master	8	(9.5)	147	(70.0)	155	(52.7)
PhD	−	(−)	3	(1.4)	3	(1.0)
Discipline^a^						
Psychology	3	(3.6)	94	(44.8)	97	(33.0)
Nursing/social educator	29	(34.5)	48	(22.9)	77	(26.2)
Medicine	2	(2.4)	46	(21.9)	48	(16.3)
Social worker	19	(22.6)	10	(4.8)	29	(9.9)
Other	13	(15.5)	3	(1.4)	16	(5.4)
Auxiliary nurse	11	(13.1)	1	(0.5)	12	(4.1)
Physiotherapy	7	(8.3)	5	(2.4)	12	(4.1)

Sample sizes vary slightly because of missing data

^a^Significant differences between primary and specialized care: χ^2^ test, *P* ≤ 0.001

### Measures

The present study focuses on the EBPAS [7], which is a well-established 15-item measure that generates four scales: 1) Intuitive appeal (four items), refers to whether practitioners will use an innovation if it is attractive, gives meaning, can be used correctly, or is being used by colleagues who are pleased with it; 2) Requirements (three items), refers to whether practitioners will use the innovation if it is requested by the service, supervisor or by agency mandates; 3) Openness to change (four items) is the degree to which practitioners are willing to try new interventions; 4) Divergence (four items) refers to whether practitioners experience research-based interventions as not clinically useful and less important than clinical experience (reverse scored on the EBPAS total score). The total scale measures practitioners’ attitudes towards implementing EBP [12]. A five-point Likert scale is used to assess degree of agreement with a given statement (0 = not at all to 4 = to a very great extent). Higher mean scores indicate more favourable attitudes. Permission from the author was obtained to use the EBPAS questionnaire. The scale was translated into Norwegian by the first author. Conceptual equivalence was emphasized. The last author back-translated the scale and compared this with the original version together with the first author. It was concluded that no significant differences appeared during the translation process.

Practitioners’ demographics were assessed through self-reported age, sex, years of experience in current profession, education length, and profession.

### Data analysis

To examine the psychometric properties of the EBPAS, Cronbach’s alpha and the within-clinic intraclass correlation coefficients (ICC) were calculated. An exploratory factor analysis was done using maximum likelihood and oblimin rotation using the psych R-package [30]. Four factors were extracted to see if the factors corresponded to the four subscales. Factor loadings below 0.32 were not shown [31]. A CFA was conducted using the psych R-package [30] to examine whether the four proposed factors and the higher-order factor in the EBPAS structure remained valid in this sample. A grouped CFA, or measurement invariance analysis, was also conducted to investigate whether the factor structure differed between specialized and community mental health services.

To examine the differences in attitudes among practitioners, the analyses were run using SPSS (Version 21). A one-sample *t*-test was used to compare the mean of the EBPAS total and the four factors in the Norwegian sample with an earlier study of mental health practitioners in the United States [12]. Correlational matrix and multivariate regression models were used to evaluate the association between practitioner demographic characteristics and the EBPAS. To handle three missing values in the practitioners’ experience variable, we used the pairwise deletion option in SPSS; *t*-tests were used to test differences in EBPAS scores between community and specialized care. Levene’s tests were used to decide whether the *t*-tests should be performed under the assumption of equal variances.

## Results

### Psychometric properties

Table 2 shows overall means and standard deviations, alpha values, ICC values, and item loadings for each of the scales. Reliability coefficients for the factors ranged between 0.64 and 0.88. Contrary to earlier research, item 14 loaded on the requirements factor (*b* = 0.43) instead of the appeal factor. The alpha value of the appeal factor showed only a small improvement if the item was deleted (α = 0.75 compared to α = 0.74). Based on earlier research supporting the original factor structure [10, 12], and due to the fact that the noise from the item was assumed to be limited, the item was retained in the appeal factor for further analyses. Regarding ICC, item 13 (“it was required by your state”) had an ICC value of 0.155. It may be that the translation of the item was weak, as it refers to municipalities rather than to authorities. The alpha value showed no improvements in the requirements factor if item 13 was deleted, so it was kept in the analyses. The remaining ICC values ranged between 0.00 and 0.05, indicating a relatively small degree of dependency among practitioners within the same clinic.

**Table 2:** EBPAS subscale and item means, standard deviations, Cronbach’s alpha, factor loadings from exploratory factor analysis, and intraclass correlation coefficient

EBPAS subscales and total	M	SD	α	1	2	3	4	ICC
1. Requirements	2.50	0.90	0.88					0.10
12 Agency required	2.72	0.93		0.81				0.05
11 Supervisor required	2.43	0.94		0.84				0.04
13 State required	2.33	1.13		0.87				0.16
2. Appeal	3.03	0.57	0.74					0.00
10 Makes sense	3.21	0.65			0.82			0.01
9 Intuitively appealing	2.90	0.76			0.82			0.03
14 Colleagues happy with intervention	2.84	0.79		0.43				0.01
15 Enough training	3.15	0.81			0.44			0.00
3. Openness	2.65	0.70	0.82					0.00
2 Will follow a treatment manual	2.78	0.83				0.72		0.00
4 Therapy developed by researchers	2.73	0.83				0.70		0.00
1 Like new therapy types	2.67	0.90				0.77		0.01
8 Therapy different than usual	2.45	0.92				0.65		0.02
4. Divergence	1.09	0.62	0.64					0.00
5 Research based treatment not useful	0.60	0.80					0.58	0.01
7 Will not use manualized therapy	0.60	0.86					0.61	0.00
6 Clinical experience more important	1.87	0.90					0.47	0.00
3 Know better than researchers	1.29	0.97					0.59	0.00
EBPAS total	2.77	0.47	0.81					0.02

Note. Factors loading < 0.32 are not shown

**Fig. 1:**
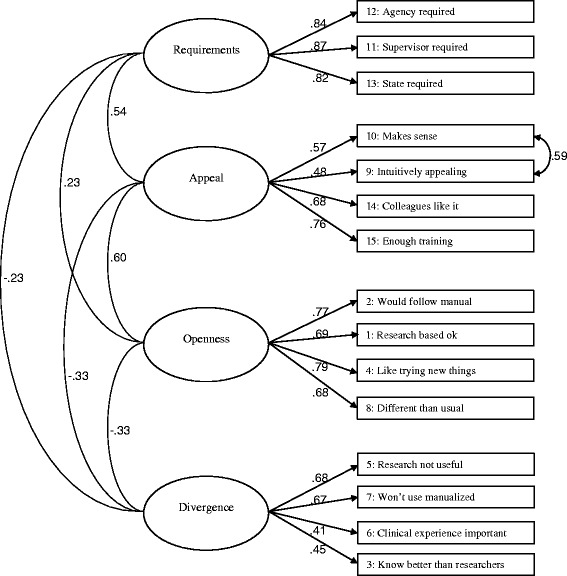
Confirmatory factor analysis model of the EBPAS. n = 294, χ^2^ (83) = 205.615, *P* < 0.001; RMSEA = 0.071; 90% CI, 0.059–0.083; CFI = 0.924; TLI = 0.903; SRMR = 0.065. All factor loadings are significant at *P* < 0.01

**Fig. 2:**
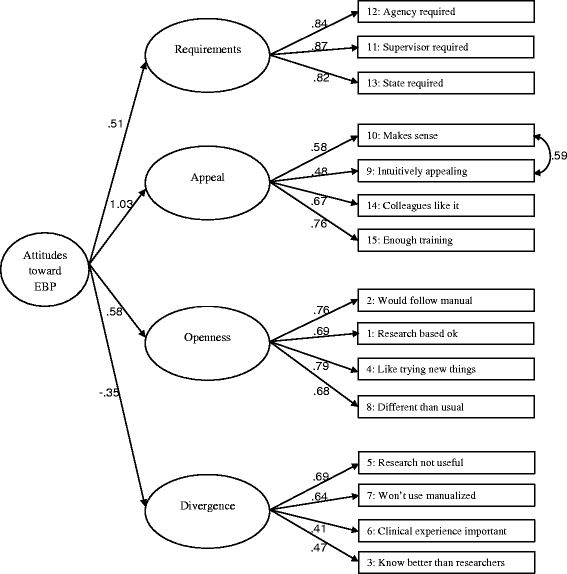
Higher-order confirmatory factor analysis model of the EBPAS. n = 294, χ^2^ (85) = 212.653, *P* < 0.001; RMSEA = 0.071; 90% CI, 0.060–0.084; CFI = 0.921; TLI = 0.902; SRMR = 0.068. All factor loadings are significant at *P* < 0.01

According to an acceptable range for model fit for CFA [32], the four factor model did not meet the criteria (χ^2^ (84, N = 294) = 285.705, *P* < 0.001; RMSEA = 0.081; 90% CI, 0.079–0.102; CFI = 0.874; TLI = 0.843; SRMR = 0.081). Model modification indices showed that model fit would improve if the residuals for item 9 (“it was intuitively appealing”) and 10 (“it made sense to you”) were allowed to correlate, which is similar to earlier factor analysis of the EBPAS [12, 33]. The revised model fit the data adequately (χ^2^ (83, N = 294) = 205.615, *P* < 0.001; RMSEA = 0.071; 90% CI, 0.059–0.083; CFI = 0.924; TLI = 0.903; SRMR = 0.065). Factor loadings ranged from 0.41 to 0.87 and factor intercorrelations ranged from 0.23 to 0.61. All factor loadings were statistically significant (Fig. 1). Similar to the four factor model, the higher-order factor did not meet the criteria (χ^2^ (86, N = 294) = 291.384, *P* < 0.001; RMSEA = 0.090; 90% CI, 0.079–0.102; CFI = 0.872; TLI = 0.844; SRMR = 0.085), but improved adequately when items 9 and 10 were correlated (χ^2^ (85, N = 294) = 212.653, *P* < 0.001; RMSEA = 0.071; 90% CI, 0.060–0.084; CFI = 0.921; TLI = 0.902; SRMR = 0.068). Factor loadings ranged from 0.35 to 1.04. All factor loadings were statistically significant (Fig. 2).

Due to the ICC value of item 13, measurement invariances were examined between specialized and community mental health services. The four factor model with a correlation of items 9 and 10 was used. When all parameters were constrained to be equal in the two groups, the model was adequate (χ^2^ = 420.126, *P* < 0.001; CFI = 0.869; TLI = 0.859; SRMR = 0.095). When item 13 was allowed to load differently in the two groups, the model showed a small improvement (χ^2^ = 413.894, *P* < 0.001; CFI = 0.872; TLI = 0.861; SRMR = 0.097). Item 13 loaded higher on requirements among practitioners in community services than in specialized services (*P* = 0.013).

### Differences in attitudes

When comparing the Norwegian and United States [12] samples of mental health practitioners’ attitudes towards EBP, results suggest there were significant differences on appeal (*M* = 3.03 vs. *M* = 2.91), *t* (293) = 3.469, *P* = 0.001), openness (*M* = 2.65 vs. *M* = 2.76), *t* (293) = −2.580, *P* = 0.01), and divergence (*M* = 1.09 vs. *M* = 1.25), *t* (293) = −4.416, *P* = 0.000). There were no significant differences on the EBPAS total (*M* = 2.77 vs. *M* = 2.73), *t* (293) = 1.524, *P* = 0.13) or requirements (*M* = 2.50 vs. *M* = 2.41), *t* (293) = 1.668, *P* = 0.09).

In terms of individual factors that predict attitudes towards EBPs, years of experience and age were associated with all of the subscales and the EBPAS total in the correlational matrix, indicating that more experience and older age were associated with more negative attitudes towards EBP (Table 3). In addition, being female was associated with greater positive attitude in general, greater willingness to adopt given the appeal of the intervention, and more openness towards EBPs. Higher educational attainment was associated with lower scores on the requirement scale, indicating that the higher the educational level, the less likely to adopt an EBP if it was required. Because there were high correlations between practitioners’ age and clinical experience (*r* = 0.79, *P* < 0.01), and health service and educational level (*r* = 0.60, *P* < 0.01), age and educational level were dropped in the subsequent regression analyses to avoid multicollinearity. When controlling for sex and health service, multiple regression analyses confirmed the presence of strong associations between practitioners’ years of experience and all of the EBPAS scales (Table 4). Furthermore, when controlling for experience and health service, multiple regression analyses confirmed the presence of strong associations between sex and EBPAS total and appeal, in addition to a weak association between sex and openness (Table 4).

**Table 3:** Descriptive statistics and correlation matrix of the demographic characteristics and the EBPAS

Number	Variable	*M*	*SD*	1	2	3	4	5	6	7	8	9	10
294	1. Sex	0.72	0.45	–									
291	2. Years of experience	13.9	9.89	0.03	–								
289	3. Age	44.93	10.81	−0.04	0.79**	–							
293	4. Education level	2.49	0.63	−0.07	−0.21**	−0.25**	–						
294	5. Health service	0.29	0.45	−0.02	−0.01	0.10	−0.60**	–					
294	6. Appeal	3.03	0.57	0.17**	−0.22**	−0.26**	0.08	0.06	–				
294	7. Requirements	2.50	0.90	0.08	−0.15**	−0.13*	−0.20**	0.23**	0.39**	–			
294	8. Openness	2.65	0.70	0.13*	−0.16**	−0.15*	0.10	0.07	0.49**	0.19**	–		
294	9. Divergence	1.09	0.62	−0.07	0.17**	0.20**	−0.04	0.03	−0.18**	−0.16**	−0.17**	–	
294	10. EBPAS total	2.77	0.47	0.16**	−0.26**	−0.26**	−0.02	−0.15*	0.73**	0.72**	0.67**	−0.53**	–

Note. Sex is coded 0 = male, 1 = female. Education level is coded 1 = Lower, 2 = Bachelor, 3 = Master, 4 = PhD Health service is coded 0 = specialized care, 1 = primary care

*P < 0.05 **P < 0.01 (two-tailed)

**Table 4:** Regression of EBPAS scale scores on years of experience controlling for sex and health service

		EBPAS total (*R* ^*2*^ = 0.12)				Appeal (*R* ^*2*^ = 0.08)			Requirements (*R* ^*2*^ = 0.09)			Openness (*R* ^*2*^ = 0.05)			Divergence (*R* ^*2*^ = 0.04)	
*n* ^*a*^	*Characteristics*	B	*SE*	*p*	β	*SE*	*P*	β	*SE*	*p*	β	*SE*	*p*	β	*SE*	*p*
291	Years of experience	−0.261	0.003	0.000	−0.225	0.003	0.000	−0.155	0.005	0.007	−0.163	0.004	0.005	0.171	0.004	0.003
294	Sex	0.170	0.057	0.002	0.177	0.071	0.002	0.088	0.112	0.121	0.131	0.090	0.023	−0.072	0.079	0.214
294	Health service	0.150	0.057	0.007	0.059	0.071	0.294	0.235	0.112	0.000	0.074	0.089	0.202	0.026	0.079	0.653

Note: Sex is coded 0 = male, 1 = female. Health service is coded 0 = specialized care 1 = primary care

^*a*^ Due to missing values cases have been excluded pairwise

(two-tailed)

When comparing attitudes between practitioners in specialized (*M* = 2.37, *SD* = 0.90) and primary care (*M* = 2.83, *SD* = 0.80), there was a significant difference on the requirement scale, (*t* (292) = 4.102, *P* < 0.001; Cohen’s *d* = 0.54). There was also a small significant difference between specialized (*M* = 2.73, *SD* = 0.46) and primary care (*M* = 2.88, *SD* = 0.47) on the EBPAS total (*t* (292) = 2.539, *P* = 0.012; Cohen’s *d* = 0.32). There were no differences on the appeal, openness and divergence scales. Equal variances were accordingly assumed for all the scales. In addition, when controlling for experience and sex, multiple regression analyses confirmed the presence of strong associations between health service and EBPAS total and requirements.

## Discussion

The current study provided evidence about the potential usefulness of the Norwegian version of the EBPAS in mental health services by investigating the psychometric properties of EBPAS scores in a sample of 294 mental health practitioners from both primary and specialized care. The study also confirmed associations between practitioners’ attitudes towards implementing EBPs and several demographic characteristics. This can be useful in the search for better strategies to implement new interventions in service settings.

Specifically, the study results supported the structure of four specific factors and a higher-order factor of the EBPAS, consistent with prior research [7, 12]. However, item 14 loaded on the requirement subscale instead of the appeal subscale. Two other psychometric studies of the EBPAS have also reported low factor loadings for item 14. Aarons [7] reported a factor loading of 0.48, and Melas et al. [10] reported a factor loading of 0.43. This may help explain why the CFA did not show perfect fit. This item distinguishes itself from the other items on the appeal factor in that it assesses how other people influence the individual’s attitudes towards EBPs (similar to the items on the requirements factor), rather than the individual’s own experiences. This might have influenced how the responders answered the question. Future research should address the issue of weak factor loadings in the EBPAS.

The sample of Norwegian practitioners showed no differences in attitudes towards EBP on EBPAS total compared to a nationwide sample drawn from the United States [12]. Accordingly, it may be assumed that Norwegian and United States mental health practitioners share many of the similar attitudes towards implementing EBP. However, differences on the appeal, openness and divergence subscales may imply cultural differences between the two nations, in addition to differences in the organization of health services and education of mental health practitioners. In terms of individual factors that have been examined as predictors of attitudes towards EBPs, years of experience seems to be congruent with most of the earlier studies [10, 21]. The more experienced the practitioner, the more negative towards EBPs. This indicates strong findings and may imply that practitioners gain more confidence on their own skills and become less curious towards new methods as they gain experience. Regarding other individual factors, earlier findings on age were initially mixed, but this study supported the findings of others that found younger practitioners to be more likely to score higher on the EBPAS total [10, 17] and lower on divergence [25]. Results on sex supported the majority of findings, namely that females score higher on appeal and the EBPAS total [12, 23]. In addition, the findings were also expanded to account for openness. It seems that female practitioners in general are more open to EBPs if these are appealing. Findings on education level did not support the many prior studies that showed associations with higher scores on appeal and the EBPAS total [22, 24]. It may be that there are cultural differences involved, and that Norwegian practitioners with higher education are more autonomous and sceptical towards EBPs than their colleagues elsewhere. The fact that higher education was associated with lower scores on requirement was confirmed. Due to differences in sample sizes and sample characteristics of the studies mentioned, it may be challenging to compare results. This also concerns findings based on regression analyses containing multiple variables [7, 12]. Due to the risk of multicollinearity, it is difficult to decide the unique contribution of every variable.

In terms of differences between primary and specialized care, the results showed that primary care employees were significantly more willing than practitioners in specialized care to use an EBP innovation if it was required by the organization. This indicates primary care practitioners are more strongly affected by organizational requirements than their colleagues in specialized care. According to Ajzen’s [5] theory, they accept the social pressure put on them (subjective norms). This may be explained by the fact that primary care practitioners have fewer formal guidelines imposed on them [27], and that they are perceived as being less bureaucratic [7] and under less stress [21]. Therefore, they can decide whether or not they will perform the behaviour. Conversely, practitioners in specialized care are subject to more formalized guidelines [27]. According to Ajzen’s [5] theory, feelings of pressure and decreased flexibility can lead to a decreased sense that practitioners are capable of implementing innovations. Because this study did not examine practitioners’ feelings of stress and inflexibility, which would have strengthened these assumptions, further research should study these associations.

The measurement invariance analysis showed an inaccuracy on item 13 between practitioners in primary and specialized health services. Thus, it is undeterminable whether there is an actual difference in requirements or if the factor measures different concepts. Of note, in addition to differences in the requirement scale scores, primary and specialized care also differed significantly on education level and discipline (Table 1). Differences in requirements may therefore be a result of differences in education level as well as of organizational factors. This may indicate a stronger sense of autonomy and integrity on the part of the specialized practitioners. Being less willing to use an innovation when required by the organization may indicate that the doctors and psychologists in specialized care rely more on autonomous decision-making than the nurses, social educators and practitioners in other primary care disciplines. Ultimately, this alleged professional structure and culture may present different challenges for those seeking to encourage practitioners to implement an EBP.

The current study had some strengths and limitations. To our knowledge, no other study has examined attitudes towards implementing EBPs by using the EBPAS in a Norwegian context. The recruitment strategy covered mental health practitioners in both primary and specialized care in the largest catchment area of a health authority in Norway. Although the response rate was adequate, the sample represents practitioners within the same geographical area and may not be generalizable to practitioners in other areas. Because information about the non-respondents were not available, it was not possible to compare them to the respondents to examine a potential non-response bias. Furthermore, independent variables in the regression analyses only explained between 3% and 10% of the variance of the different factors. This indicates that other practitioner and organizational factors not measured in this study likely play an important role in practitioners’ attitudes towards implementation of EBP. Lastly, we did not identify practitioners’ earlier experience with EBP. The EBPAS measures general attitudes, and therefore we do not know what practice respondents had in mind when they filled in the questionnaire.

This study has added to the knowledge about the EBPAS in general and for Norway in particular. Additional research is needed to further establish the factor structure of the Norwegian version of the EBPAS. Future studies should also more thoroughly examine differences by level of experience and profession, as well as other individual and organizational characteristics that may influence attitudes. More research is also needed on the attitude–behaviour relationship, and whether or not attitudes predict the adoption, implementation and sustainment of EBPs.

## Conclusions

The EBPAS is likely to be a useful tool in future research as well as in clinical practice. Screening for attitudes towards EBP prior to implementation may indicate which attitudes are central among the participants. This may help organizations consider specific steps to influence practitioner attitudes as necessary, thereby enabling improved tailoring of implementation efforts.

## Abbreviations

EBPs, evidence-based practices; EBPAS, Evidence-Based Practice Attitude Scale; ICC, intraclass correlation coefficients; CFA, confirmatory factor analysis; RMSEA, root mean square error of approximation; CI, confidence interval; CFI, comparative fit index; TLI, Tucker–Lewis index; SRMR, standardized root mean square residual
